# Towards a global sustainable development agenda built on social–ecological resilience

**DOI:** 10.1017/sus.2023.8

**Published:** 2023-04-24

**Authors:** Murray W. Scown, Robin K. Craig, Craig R. Allen, Lance Gunderson, David G. Angeler, Jorge H. Garcia, Ahjond Garmestani

**Affiliations:** 1 Lund University Centre for Sustainability Studies (LUCSUS), Lund University, Lund, Sweden; 2 University of Southern California Gould School of Law, Los Angeles, CA, USA; 3 Center for Resilience in Agricultural Working Landscapes, School of Natural Resources, University of Nebraska-Lincoln, Lincoln, NE, USA; 4 Department of Environmental Sciences, Emory University, Atlanta, GA, USA; 5 Department of Aquatic Sciences and Assessment, Swedish University of Agricultural Sciences, Uppsala, Sweden; 6 School of Natural Resources, University of Nebraska-Lincoln, Lincoln, NE, USA; 7 Brain Capital Alliance, San Francisco, CA, USA; 8 IMPACT, The Institute for Mental and Physical Health and Clinical Translation, Deakin University, Geelong, Victoria, Australia; 9 Universidad de Los Andes, School of Management, Bogota, Colombia; 10 Office of Research and Development, US Environmental Protection Agency, Gulf Breeze, FL, USA; 11 Utrecht Centre for Water, Oceans and Sustainability Law, Utrecht University, Utrecht, The Netherlands

**Keywords:** earth systems (land; water; and atmospheric), ecology and biodiversity, natural resources (biological and non-biological), policies, politics and governance

## Abstract

**Non-technical summary.:**

The United Nations’ sustainable development goals (SDGs) articulate societal aspirations for people and our planet. Many scientists have criticised the SDGs and some have suggested that a better understanding of the complex interactions between society and the environment should underpin the next global development agenda. We further this discussion through the theory of social–ecological resilience, which emphasises the ability of systems to absorb, adapt, and transform in the face of change. We determine the strengths of the current SDGs, which should form a basis for the next agenda, and identify key gaps that should be filled.

**Technical summary.:**

The United Nations’ sustainable development goals (SDGs) are past their halfway point and the next global development agenda will soon need to be developed. While laudable, the SDGs have received strong criticism from many, and scholars have proposed that adopting complex adaptive or social–ecological system approaches would increase the effectiveness of the agenda. Here we dive deeper into these discussions to explore how the theory of social–ecological resilience could serve as a strong foundation for the next global sustainable development agenda. We identify the strengths and weaknesses of the current SDGs by determining which of the 169 targets address each of 43 factors affecting social–ecological resilience that we have compiled from the literature. The SDGs with the strongest connections to social–ecological resilience are the environment-focus goals (SDGs 2, 6, 13, 14, 15), which are also the goals consistently under-prioritised in the implementation of the current agenda. In terms of the 43 factors affecting social–ecological resilience, the SDG strengths lie in their communication, inclusive decision making, financial support, regulatory incentives, economic diversity, and transparency in governance and law. On the contrary, ecological factors of resilience are seriously lacking in the SDGs, particularly with regards to scale, cross-scale interactions, and non-stationarity.

**Social media summary.:**

The post-2030 agenda should build on strengths of SDGs 2, 6, 13, 14, 15, and fill gaps in scale, variability, and feedbacks.

## 1. Introduction

The pace and scale of environmental change has accelerated so rapidly that global stewardship and governance for sustainable development is of utmost importance (Folke et al., 2021). The alleviation of poverty, reduction of inequalities, provisioning of food, quality education, and energy access to 8 billion people involves trade-offs among the ecosystem services upon which citizens and societies ultimately depend. In response to these global challenges, most of the world has committed to pursuing the 17 United Nations’ sustainable development goals (SDGs) and their 169 targets (UN, 2015). The SDGs reflect the triple bottom line for people, profit, and the planet and are a novel approach to global governance through goal setting (Biermann et al., 2017).

While the goals are nominally co-equal in importance, mounting evidence shows that social and economic goals are prioritised over environmental ones (Craig & Ruhl, 2019; Eisenmenger et al., 2020; Forestier & Kim, 2020) and that achievement of the SDGs will not necessarily prevent environmental destruction (Zeng et al., 2020). Indeed, some have argued that the SDGs reinforce an economic growth paradigm and will lead to further unwanted environmental change, essentially counteracting their intended transformation to sustainability (Eisenmenger et al., 2020).

Critics of the SDGs have argued that the goals were formulated through political negotiation rather than derived from an integrative theoretical framework of sustainable development (Eisenmenger et al., 2020; le Blanc, 2015). Attempts at triangulating among partial environmental, economic, and social theories are unlikely to coalesce into governance mechanisms and political priorities that can achieve a triple bottom line. Specifically, without a recognition of the environment’s fundamental role in human well-being and an understanding of social–ecological system (SES) function, the achievement of *social* goals and targets for local settings over short time frames does not guarantee sustainability for the *biosphere* over the long term (Folke et al., 2016).

The SDGs are beyond their midway point and negotiations will soon need to begin to decide the global development agenda that will succeed them – a process that will undoubtedly benefit from an SES perspective (Berkes & Folke, 1998; Folke et al., 2016; Reyers et al., 2022, 2018; Selomane et al., 2019). While scholars have for some time made calls to build development agendas consistent with our understandings of SESs, the contribution to sustainable development from the theory of social–ecological resilience, in particular, is only just being explored (Elmqvist et al., 2019; Reyers et al., 2022). This discussion is especially pertinent given the call for ‘climate-resilient development’ in the latest report of the Intergovernmental Panel on Climate Change Working Group II (IPCC, 2022), as well as the sprinkling of goals to enhance ‘resilience’ throughout the SDGs themselves. Such a foundation requires a clear understanding of the features of social–ecological resilience and how those system features are supported or obscured by the current sustainable development agenda (Reyers et al., 2022).

Resilience is an emergent property of SESs that describes how much disturbance a system can withstand without shifting to a new configuration with a different set of processes, structures, and feedbacks (Allen et al., 2019). The theory addresses the surprising and unpredictable dynamism of complex systems of humans and their environments, accounts for the capacity of SESs to learn, adapt, and transform, and describes non-linear scaling and cross-scale interactions of systems moving through multi-scale adaptive cycles (i.e. panarchy) as its basic model (Gunderson et al., 2022; Gunderson & Holling, 2002). Social–ecological resilience concepts were proposed and tested in coupled human and natural systems (Folke et al., 2016) and imply that social and economic elements cannot be decomposed or separated from a complex and dynamic environment (Folke et al., 2016, 2021). Social–ecological resilience literature demonstrates that governance must account for scale, cross-scale interactions, and SES dynamics (Folke, 2006; Gunderson & Holling, 2002; Holling, 1973) even in pursuit of static goals. Such system dynamics and cross-scale interactions make it impossible for a managed system to maintain a desired goal state for any length of time (Angeler et al., 2020).

The concept of social–ecological resilience, as originally developed by Holling (1973), has been diluted through time, and has lost focus upon its core underpinnings: the multi-scale processes and structures that define linked systems of humans and their environments. Here, we refocus research on social–ecological resilience to this core conception, which applies to dynamics in linked social, ecological, and economic systems. This refocus on Holling’s perspective ensures that SES research is based upon a sound foundation that accounts for the essential role of biophysical systems in social–ecological resilience.

An important question for building the next global development agenda on resilience thinking, therefore, is the extent to which the current SDGs incorporate critical aspects of SES dynamism as revealed through social–ecological resilience research. Nevertheless, critical analysis of global sustainable development frameworks from a social–ecological resilience perspective is limited. A recent review by Reyers et al. (2022) highlighted six shifts towards resilience thinking that, if implemented, will contribute to future sustainable development: from capitals to capacities, from objects to relations, from outcomes to processes, from closed to open systems, from generic interventions to context sensitivity, and from linear to complex causality. Earlier, Selomane et al. (2019) determined the limited extent to which the SDG indicators account for five key features of SESs that relate to sustainable development: feedbacks, resilience, heterogeneity, nonlinearity, and cross-scale dynamics. Here we dive deeper into the key factors that affect the resilience of SESs and determine the extent to which they are accounted for in the current SDGs. The intersection between social–ecological resilience and the SDGs should serve as a starting point for building their next iteration soundly on theory, and the gaps should be filled where SDGs miss key factors affecting resilience. It is our identification of the features of social–ecological resilience that are supported or obscured by the SDGs that advances on previous contributions.

To assess the intersection between social–ecological resilience and the SDGs, we analyse the 169 SDG targets against a suite of 43 biophysical, social, and economic factors that affect social–ecological resilience (see Section 2 for details). Our comparison reveals critical differences between the SDGs and social–ecological resilience insights with respect to management and governance of SESs that operate within and interact across multiple scales. It is beyond our scope, and indeed will require years of inclusive deliberation, to formulate an explicit development agenda based on SES resilience to replace the SDGs; yet, our findings illuminate where to start. Future development goals should account for scale and cross-scale interactions, slow changes in driver variables, underlying non-stationarity, and possible tipping points, particularly as the impacts of climate change accelerate SES dynamism. The next global sustainable development agenda can build on the strengths of the current SDGs in terms of social–ecological resilience – namely, in the environment-focused goals (SDGs 2, 13, 14, 15) – and should be wary of the implications of missing key factors that affect the resilience of SES. Here we further the discussion on why.

## 2. Methods

We conducted a qualitative assessment of the extent to which the 169 SDG targets align with key factors contributing to social–ecological resilience. We focused on SDG targets instead of indicators to reduce the ‘slippage’ away from potentially transformative goals when translated into targets and further into indicators, which has been well documented (Fisher & Fukuda-Parr, 2019; Fukuda-Parr & McNeill, 2019). First, we derived from the literature a list of 43 system factors across 10 biophysical, social, and economic dimensions that are known to influence social–ecological resilience (Tables 1–3). Next, the 169 SDG targets were assessed to determine which of the 43 resilience factors they aligned with. This alignment was based on the official descriptions of the SDG targets and was carried out through debate and expert judgement among the authors. Any SDG target that implicates a resilience factor was considered to align with that factor (Tables 1–3). For example, SDG target 14.3 is to ‘[m]inimize and address the impacts of ocean acidification…’, which is a slow variable affecting the resilience of marine SESs. Thus, SDG target 14 aligns with the resilience factor of ‘identification of slow variables’ in the biophysical dimension of resilience (Tables 1–3). We were favourable to the SDGs in that even weak connections with resilience factors were considered to align, so our assessment is a ‘best case’ scenario of how well the SDGs account for social– ecological resilience. In addition to those targets aligned with a particular resilience factor in Tables 1–3, we also include target 13.1 (‘Strengthen resilience and adaptive capacity to climate-related hazards and natural disasters in all countries’) because it has resilience at its core although it does not align with a specific factor.

Following alignment of the 169 SDG targets with the 43 social–ecological resilience factors, we determined which SDGs best encompass core, foundational social–ecological resilience factors (Holling, 1973) by calculating the fraction of targets within each SDG that aligned with any resilience factor. We also tabulated which resilience factors and incorporated (at least in part) into the SDGs. The first of these analyses tells us where the strengths lie in terms of social–ecological resilience in the current SDGs; the second tells us where the current gaps are in terms of building a global sustainable development agenda on social–ecological resilience.

## 3. Results

**Figure 1.**
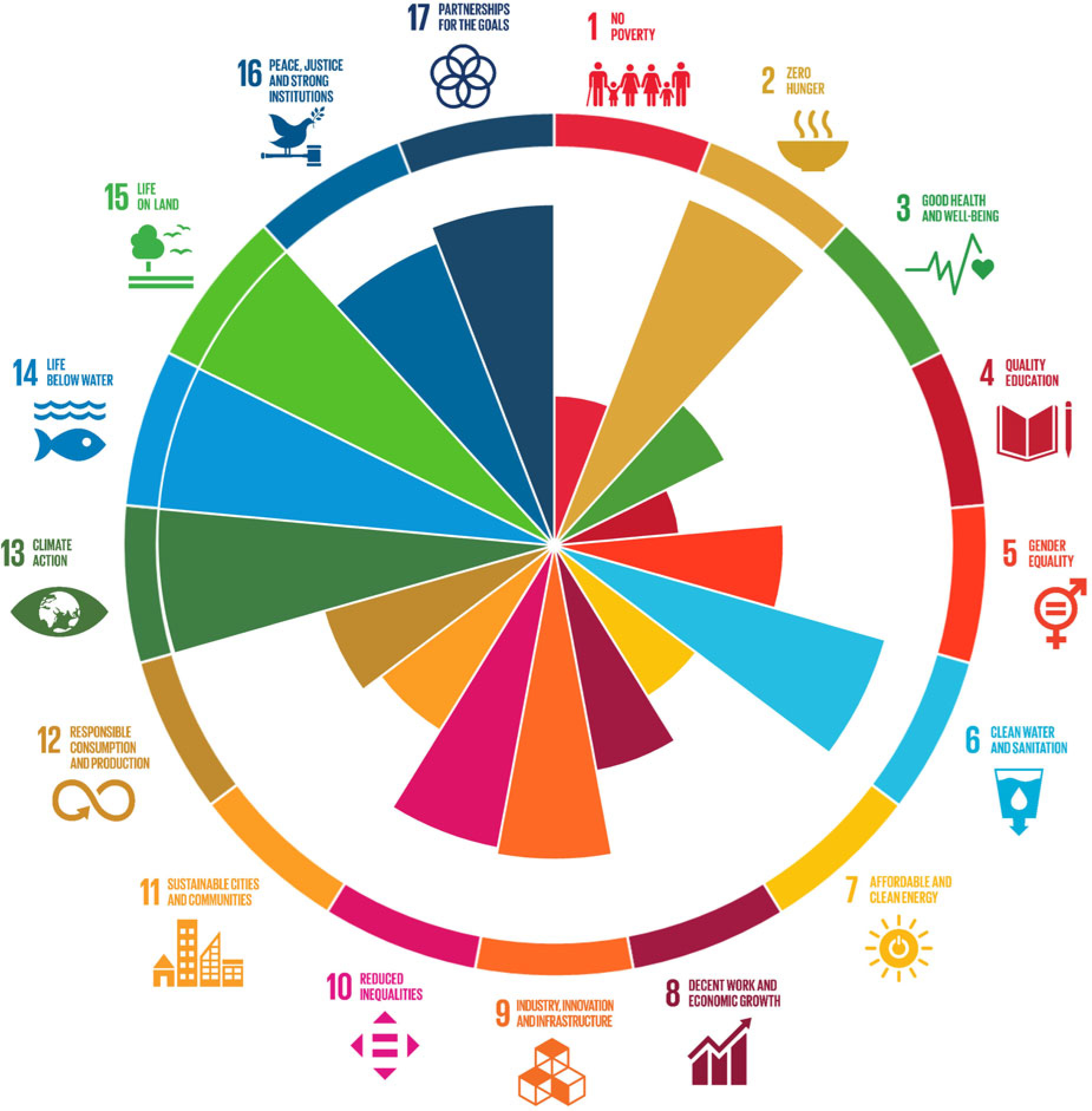
Performance of each SDG in terms of targets aligning with social–ecological resilience factors. Bars represent the fraction of targets within each SDG that align with one or more of the 43 resilience factors. All of the environmental targets within the SDGs of climate (SDG 13), oceans (SDG 14), and life on land (SDG 15) align with factors of resilience (although the resilience connections to target 13.1 are implicit and general rather than specific), as do most of the targets for zero hunger (SDG 2) and clean water (SDG 6). Good governance (SDG 16) and partnerships (SDG 17) also align well with social–ecological resilience. The weakest SDGs in terms of their cognisance of social–ecological resilience are the traditional development goals of no poverty (SDG 1), good health and wellbeing (SDG 3), and quality education (SDG 4).

Our comparison of the SDG targets against the factors that contribute to social–ecological resilience yields four primary results. First, several of the SDGs have significant alignment with social–ecological resilience in terms of their targets. Perhaps unsurprisingly, given social–ecological resilience’s origins in ecology, the SDGs with the strongest links to social–ecological resilience are: the environmental SDGs (SDGs 13, 14, and 15); SDG 6 – Clean Water, which is partly an environmental goal; and SDG 2 – Zero Hunger, which has strong ties to agriculture, fishing, and aquaculture and hence to the environment (Figure 1). Strong examples of the ways in which these SDGs acknowledge factors of social–ecological resilience include: the protection of biodiversity; the conservation of different ecosystems (e.g. mountains, coastal areas) in light of their spatial heterogeneity; the management, protection, and restoration of ecosystem services; the attention to slow variables such as ocean acidification and desertification; and the integration of climate and environmental change into regional planning and finance. However, these same SDGs with strong connections to resilience factors are also consistently under-prioritised in the implementation of the agenda (see Craig and Ruhl, 2019; Custer et al., 2018; Forestier and Kim, 2020). SDG 17, Partnerships, also has strong connections to social–ecological resilience, a result of the fact that the two share emphases on multiple voices, participation, and redundancy within governance.

Second, and more intriguing, the United Nations’ current descriptions of several SDGs and targets that *should* have clear connections to social–ecological resilience often do not. Of particular interest here are the traditional development goals: SDGs 1 (No Poverty), 3 (Good Health and Well-Being), and 4 (Quality Education). All three of these SDGs are critical to social adaptive capacity, social–ecological resilience, and effective governance. Nevertheless, the targets within these goals do not explicitly reflect critical factors of social–ecological resilience (Figure 1). For example, most targets within SDG 4 are typical development targets (e.g. build schools, increase the number of teachers, ensure that all children achieve literacy and numeracy) that, although critical and laudable, do not capture other types of knowledge and learning that are necessary to effectively govern social–ecological resilience. Quality Education targets that consciously incorporate social–ecological resilience would include: the adoption of social learning frameworks into organisations and institutions at multiple scales and outside formal education (Berkes, 2009); the sharing of knowledge; and a recognition and incorporation of different forms of knowledge (e.g. indigenous knowledge) (Mastrángelo et al., 2019) that extend several steps beyond target 4.7’s ‘appreciation of cultural diversity and of culture’s contribution to sustainable development’. The failure of the current goals most pertinent to future governance to embrace critical factors of social–ecological resilience provocatively suggests that the nations developing environmental governance through pursuit of the SDGs are *not* developing the pluralistic and multi-scale governance institutions and organisations necessary for environmental governance in the Anthropocene. This failure, in turn, perhaps reveals a latent institutional resistance to acknowledging that sustainable development should be conceived of less as a *goal* (or 17 goals) than as a structured, iterative *process* that must be governed within a continually changing world.

**Figure 2.**
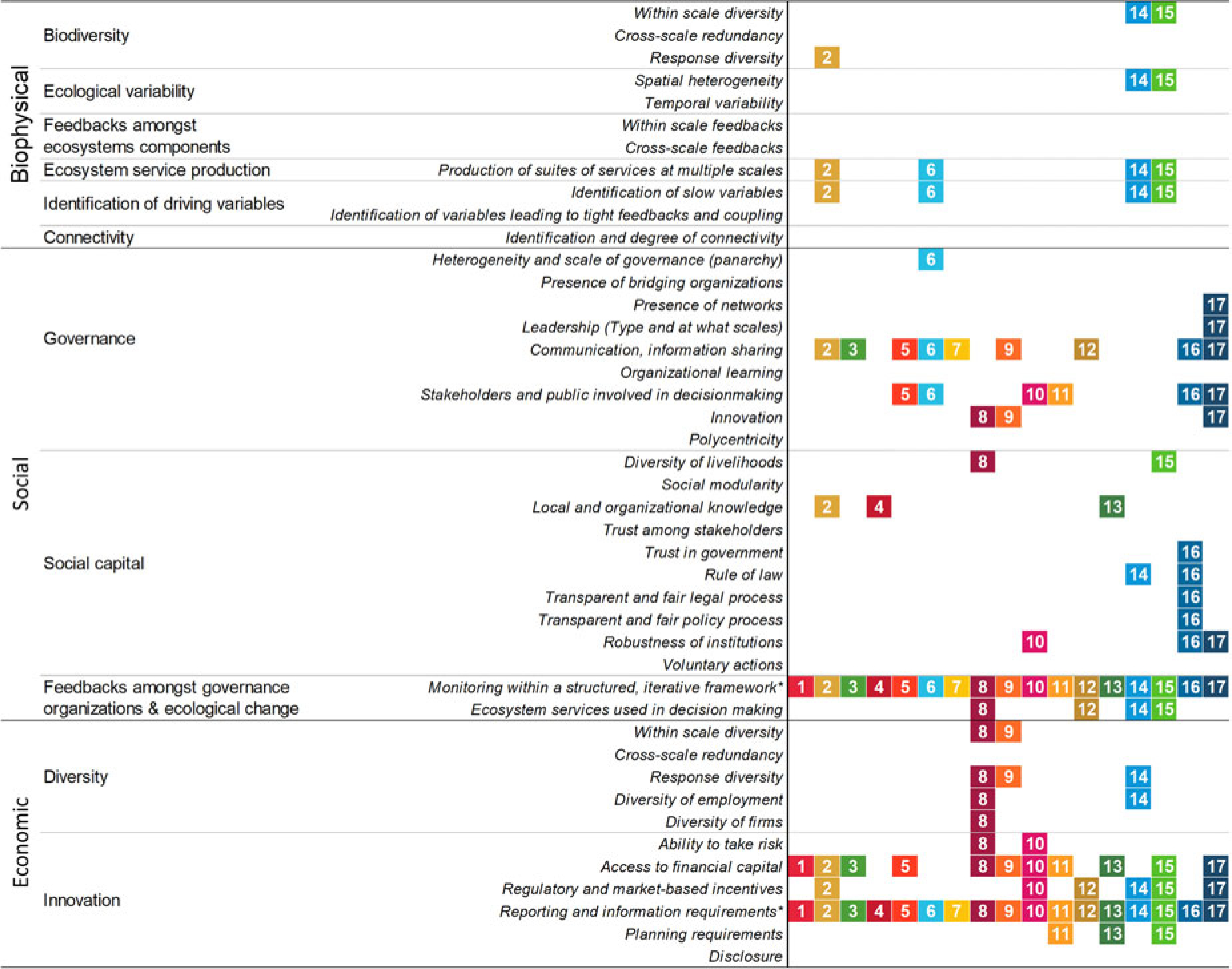
Alignment of SDG targets with 43 social–ecological resilience factors. In terms of social–ecological resilience, the SDG strengths lie in their communication, inclusive decision making, financial support, regulatory incentives, economic diversity, and transparency in governance and law. On the contrary, ecological factors of resilience are seriously lacking in the SDGs, particularly with regards to scale, cross-scale interactions, and non-stationarity. *Note: monitoring, review, and follow-up underpin the 2030 Agenda, yet strictly speaking, the framework is not structured in such a way to enable iterative learning as part of the SDGs, which is critical for managing social–ecological resilience.

Third, the SDGs as a whole fail to incorporate several key factors critical for adaptive or transformative governance of SESs (Figure 2). Thirteen of the 43 factors are completely missing from the SDGs, including cross-scale redundancy, temporal variability, ecosystem feedback indicators of any type, bridging organisations, social modularity, and trust among stakeholders. Fifteen more factors affecting social–ecological resilience are present in only one or two of the SDGs, including different types of diversity (e.g. within-scale biodiversity, economic diversity, and response diversity) and most of the social capital and governance factors that resilience scholars deem necessary for effective governance of social–ecological resilience (Craig et al., 2017; Folke, 2006; Garmestani & Benson, 2013; Gunderson & Holling, 2002). These factors are critical for facilitating the adaptability and transformability of SESs, and the omission of many could lead to the erosion of social–ecological resilience at multiple scales, including, eventually, at the global scale. For example, the social–ecological resilience of the Earth depends upon self-organisation of the many biophysical systems that manifest at multiple scales and enable the planet to withstand perturbations (Gunderson et al., 2022). This multi-scale organisation of Earth provides resilience, but it also means that change can happen quickly and unexpectedly (nonlinear change). While physical processes of Earth are often continuous and scalable, coupled human–biophysical systems are modular and multi-scale (Gunderson & Holling, 2002). Thus, accounting for scale and cross-scale interactions is critical for improving the SDGs in order to avoid disastrous consequences.

Finally, our finding that all SDGs address the key factors of monitoring and reporting (Figure 2) requires further interpretation as to whether the SDGs’ conception of monitoring meets the requirements that social–ecological resilience would demand. Clearly, monitoring is needed to evaluate system trajectory and response to interventions. Yet, monitoring alone is insufficient for sound environmental governance if the information does not lead to social learning. When dealing with dynamic SESs, a structured, iterative process for governance that incorporates monitoring information at decision points is essential for learning and sound governance (Herrmann et al., 2021). The SDG indicators used for monitoring and evaluation have already come under heavy scrutiny for their failure to capture indispensable system variables, important differences between contexts, and feedbacks between the environment and society (Reyers & Selig, 2020; Reyers et al., 2017; Szabo et al., 2016; Zeng et al., 2020), as well as for their highly contested and political nature (Fisher & Fukuda-Parr, 2019; Fukuda-Parr & McNeill, 2019). Adding to these concerns, a monitoring framework based on social–ecological resilience would include variables essential to defining system state (Reyers et al., 2017), including indicators that can give early warning of regime shifts (Folke, 2006), instead of merely monitoring progress towards static targets, as in the SDGs. In addition, because of within-scale and cross-scale interactions, the SDGs’ current emphasis on national-scale monitoring should be expanded to provide information regarding the state and functioning of SESs operating at multiple spatial and temporal scales, both sub-national and super-national (Gunderson & Holling, 2002), especially those SES that cross national borders (e.g. transnational river basins; Scown, 2020).

## 4. Discussion

Our analysis reveals that the strengths of the SDGs in terms of social–ecological resilience lie in the environment-focused goals (SDGs 2, 6, 13, 14, 15). Nevertheless, the targets as currently formulated are missing three critical components. First, the SDGs do not adequately account for scale, cross-scale interactions, and the dynamism of SESs. Second, the framework of goals and targets implies stationarity (i.e. that development can continue in a continuous fashion towards fixed end points), resulting in an inadequate monitoring process that is neither appropriately structured nor iterative – two requirements necessary to enable learning and adjustment in goals. Finally, the SDGs and targets do not address social, ecological, or governance capacity for adaptation and transformation in the face of non-stationarity and tipping points.

The only example of a strength of the current SDGs with regards to cross-scale interactions and governance is that of target 6.5 to ‘By 2030, implement integrated water resources management at all levels, including through transboundary cooperation as appropriate’. Integrated and transboundary water resources management is essential to account for cross-scale interactions in river basins. Take, for example, the case of the Mekong River Basin. The Mekong Delta is at risk of drowning largely because of land subsidence and a reduction in sediment delivery to the delta (Dunn & Minderhoud, 2022; Dunn et al., 2019; Minderhoud et al., 2020). While land subsidence is largely caused by groundwater extraction at the delta scale (Minderhoud et al., 2017), the construction of dams (often for hydroelectricity production in pursuit of sustainable development) throughout the basin drives the decline in sediment delivery (Dunn et al., 2019). While one or a few dams on tributaries may not dramatically impact basin-wide sediment flows, many dams on many tributaries add up to basin-scale effects (Schmitt et al., 2018). Without integrated transboundary water resources management (and, perhaps even with), Vietnam, where the Mekong Delta is mostly located, is at the mercy of upstream countries pursuing their own water resources development.

Our results support earlier findings that feedbacks between the environment and society are key knowledge gaps for sustainable development (Mastrángelo et al., 2019), that the SDGs have limited cognisance of scale, cross-scale interactions, and the dynamics of SESs (Selomane et al., 2019), and that the Agenda focuses on economic growth over ecological integrity (Eisenmenger et al., 2020). The current SDGs promote a fixed form of governance to achieve goals; whereas development goals built on social–ecological resilience would instead promote governance institutions and organisations that acknowledge continuous change and potential surprises, that value adaptive and transformative capacities as critical strengths in iteratively maintaining social, economic, and environmental goals over time, and that actively incorporate data collection and institutional mechanisms to enable the social learning necessary for governing SESs.

Intentionally or not, the SDGs promote governance regimes that define a relatively static end social–ecological state to be achieved – one that meets *goals* and *targets*. Targets are by definition end points to be achieved. By contrast, governance consistent with our understanding of social–ecological resilience should be dynamic and focused upon *processes* – that is, governance able to define and re-define the targets themselves in a structured, iterative framework in response to changing conditions (Garmestani & Benson, 2013; Herrmann et al., 2021). More importantly, the SDGs and targets tempt governments to think solely within their own borders rather than considering a dynamic and interactive system of systems that operates across several scales, none of which neatly match national borders (Gunderson & Holling, 2002; Scown, 2020). National governments that pursue SDGs while failing to account for social–ecological resilience are unlikely to achieve the social–ecological–economic stability and equity that they seek and run the risk of eroding the resilience of SESs at multiple scales over time, therefore putting at risk the living systems upon which all life depends.

More broadly, our study shows that the SDGs and social–ecological resilience currently embody fundamentally different worldviews. Social–ecological resilience embodies a worldview of continuous change and emphasises diversity, redundancy, adaptability, and transformability. Diversity and especially redundancy often aren’t efficient, particularly when viewed with short-term myopia, but governance for social–ecological resilience prioritises guaranteeing long-term function over a wide range of conditions rather than optimising currently desired outputs under static conditions. To make the distinction concrete, governance for forests based on social–ecological resilience seeks to ensure that some form of biodiverse forest providing multiple ecosystem services still exists a century from now, while stationarity-based development governance seeks to ensure a set yearly tonnage of pine timber over the term of the relevant political cycle (see, cf. Heilmayr et al., 2020).

Despite the claim that they are transformative, the SDGs are arguably still based on a paradigm of economic growth and efficiency under static conditions (Eisenmenger et al., 2020), rather than a worldview compatible with SES dynamism. For example, target 8.1 requires that countries ‘sustain per capita economic growth’, while target 7.3 seeks to ‘double the global rate of improvement in energy efficiency’. SESs that are managed based on static end points, growth, and efficiency may be ultimately vulnerable to both natural and human-induced disasters and unexpected events. Indeed, a focus on growth and efficiency can lead to increased resource consumption and associated environmental damage (Eisenmenger et al., 2020) – known as Jevons’ paradox (Alcott, 2005).

A sustainable system should be both in a desirable state and resilient to future intrinsic and extrinsic surprise – that is, it can withstand disturbances coming from within and outside of the system while resisting reorganisation into a new state. However, although pursuit of development goals often induces governance systems and managers to actively (and expensively) fight transformation (Angeler et al., 2020) (e.g. maintaining energy subsidies and infrastructure focused upon fossil fuels instead of transitioning to renewable energy), governance based on social–ecological resilience views those SESs on the brink of unsustainability – systems currently in a desirable state but no longer resilient to surprises – as opportunities. Managers operating under the social–ecological resilience paradigm would actively manage these systems for transformation, deliberately pushing the system beyond its resilience to foster a shift into an alternative, desirable configuration (Chaffin et al., 2016; O’Brien, 2011; Westley et al., 2011). As one example, the difference in these approaches may be critical to the sustainability of the world’s cities, which are now home to the majority of the world’s population (with many living in poverty) and are the source of the majority of greenhouse gas emissions (Elmqvist et al., 2019). In order to achieve desirable cities, decision-makers must understand and manage their social–ecological resilience – which can in many cases be undesirable in the form of poverty traps and technological ‘lock-in’ – and govern cities through transformations towards desirable configurations, fostering new transportation systems, new building and neighbourhood design, and new job opportunities, among many other components (Elmqvist et al., 2019).

Despite our findings that the strongest connections to social–ecological resilience lie in the environment-focused SDGs, nations’ priorities in implementing the current agenda promote social and economic goals ahead of environmental ones. Instead, environmental governance should reflect the reality that the Earth’s planetary biosphere is itself a large-scale complex adaptive system whose survival depends on myriad of feedbacks among physical, chemical, and biological processes operating at multiple temporal and spatial scales (Gunderson & Holling, 2002). While this large-scale system has been in a relatively stable conservation phase for roughly the last 12,000 years of the Holocene – probably *not* coincidentally encompassing the entire history of human civilisation – the planet also has tipping points at multiple scales, some of which may be in imminent danger of being crossed (Armstrong McKay et al., 2022; Hughes et al., 2013; Steffen et al., 2015, 2018 ). In contrast to the current SDGs, social–ecological resilience emphasises that the environment is the boundary of – not co-equal to – social and economic development goals (Benson & Craig, 2014; Craig & Ruhl, 2019; Gunderson & Holling, 2002) and that dynamism and tipping points at the planetary scale may put humankind at risk. For example, Steffen et al. (2018) described a set of environmental systems that have acted together to stabilise the Earth’s Holocene climate through a series of negative feedbacks. However, these systems could flip into alternative regimes, creating reinforcing positive feedbacks that cascade to the planetary scale and destabilise the climate, resulting in a so-called ‘hothouse’ Earth with potentially devastating consequences for ecosystems, societies, and economies (Steffen et al., 2018). Although the extreme example of ‘hothouse’ Earth is heavily contested and the likelihood is entirely unknown, recent updated analyses of ‘tipping elements’ in the Earth system suggest that some are at risk of being crossed even at current levels of warming (Armstrong McKay et al., 2022). The presence of such tipping points in Earth systems led scholars to push for planetary ‘must haves’ in the current set of SDGs (Griggs et al., 2013), but evidence suggests that these are not being prioritised (Craig & Ruhl, 2019; Forestier & Kim, 2020; Zeng et al., 2020).

The global influence of humans on Earth systems not only presents urgent challenges, but also the critical opportunity to steward the environment in desirable ways even at the planetary scale (Biermann et al., 2012; Folke et al., 2016; Steffen et al., 2018; Sterner et al., 2019). Governance based on social–ecological resilience acknowledges that most management targets must be subject to continual adjustment even when no obvious shocks or stressors are operating on the system of interest, a need that becomes even more critical when systems are actively changing in response to global warming, ocean acidification, and a myriad of anthropogenic stressors such as land-use change (Garmestani et al., 2020). If we are to steward the planet through these challenges via global goals such as the SDGs, then these governance-inducing global agendas must better account for multiple scales of SESs, cross-scale interactions, feedbacks between the environment and society, non-stationarity, and the social attributes of governance. However, as our analysis shows and others have argued (Mastrángelo et al., 2019; Reyers & Selig, 2020; Scown, 2020), the current SDGs do not (or only weakly in some cases) incorporate these critical factors of social–ecological resilience. We argue that the next global sustainable development agenda should at least equally prioritise the current strengths of the environment-focused goals (SDGs 2, 6, 13, 14, 15), build on the ‘good governance’ foundations of SDGs 16 and 17, fill critical gaps we have identified in those factors affecting social–ecological resilience, and design a monitoring and evaluation framework that is multi-scale, iterative, and adaptive to changing societal and environmental requirements over time.

## 5. Conclusion

Our comparison indicates that the current SDGs do not account for scale, cross-scale interactions, and the dynamics of SESs. As such, governance based on the SDGs is unlikely to be equal to the task of either achieving or maintaining those goals. Governance systems that assume stationarity and the substitutability of ecological function for social and economic prosperity must transform into institutions and organisations that prioritise the continuing functionality and resilience of the ecological components of SESs. Or, to reverse the framing, if nations want to eradicate poverty and hunger, educate their populations, and ensure economic livelihoods for their citizens well into the future, they must first assess what a changing climate, cross-scale interactions, and changing feedback loops will mean for the social–ecological resilience of their communities.

Social–ecological resilience is a body of theory derived from decades of observation and study of coupled human and natural systems, whereas the current SDGs are a set of idealistic goals that only partially address what is known about dynamic SESs. Social–ecological resilience allows for shifting approaches to governance (such as moving from fostering adaptation to encouraging transformation) and encourages assessment, learning, and re-evaluating or changing goals as necessary. The current SDGs fail to incorporate several key factors critical to the productive governance of SESs at multiple scales that are accounted for by social–ecological resilience. The next global sustainable development agenda should be a structured, iterative process that enhances learning about the dynamic *process* of sustainability through time, and nested within a resilience-based governance framework (Garmestani & Benson, 2013; Herrmann et al., 2021). Environmental governance at all scales – global, national, regional, and local – must embrace the best available understanding of SES dynamism if humanity is to navigate and thrive in the Anthropocene (Biermann et al., 2012; Garmestani et al., 2019; Sterner et al., 2019). Thus, using a social–ecological resilience lens to evaluate and reform the SDGs, increasing their capacity to promote truly effective environmental governance for the long term, helps strengthen future iterations of global sustainability agendas.

## Supplementary Material

SI

## Figures and Tables

**Table 1.** List of biophysical resilience factors that are addressed (at least in part) by SDG targets

Dimension	Resilience factor^reference^	SDGs	Targets	Rationale as to why the target aligns with the resilience factor
Biodiversity	Within scale diversity^1^	14	14.a	14.a – Acknowledges role of marine biodiversity for development, particularly SIDS and LDCs
		15	15.4	15.4 – Emphasises conservation of mountain biodiversity
			15.5	15.5 – Aims to halt loss of biodiversity and protect threatened species
			15.7	15.7 – Aims to end poaching and illegal wildlife trade, which threatens biodiversity
			15.8	15.8 – Aims to reduce the impact of invasive species, which are a driver of biodiversity loss
	Cross-scale redundancy^1^			
	Response diversity^2^	2	2.5	2.5 – Maintaining genetic diversity in seed banks across multiple levels of organisation enables future response diversity
Ecological variability	Spatial heterogeneity^3^	14	14.5	14.5 – Conservation of at least 10% of coastal and marine areas recognises importance of spatial heterogeneity
		15	15.4	15.4 – Conservation of mountain ecosystems acknowledges their spatially heterogeneity
	Temporal variability^4^			
Feedbacks amongst ecosystems components	Within scale feedbacks^5^			
	Cross-scale feedbacks^5^			
Ecosystem service production	Production of suites of services at multiple scales^6^	2	2.4	2.4 – Ensuring sustainable and resilient agriculture that maintains ecosystems recognises ecosystem service of food/fuel/fibre provisioning
		6	6.4	6.4 – Ensuring sustainable supply of freshwater implies ecosystem service of freshwater provisioning
		14	14.2	14.2 – Sustainably managing, restoring, and protecting marine and coastal ecosystems implies acknowledgement of the critical ecosystem services they produce
		15	14.7	14.7 – Increase economic benefits to small islands from fisheries, aquaculture, and tourism
			15.1	15.1 – Aims at conservation, restoration, and sustainable use of terrestrial and inland freshwater ecosystems and their services
			15.2	15.2 – Protection, sustainable management, and restoration of forests recognises their ecosystem services
			15.6	15.6 – Promotes fair and equitable benefits of the use of genetic resources provided by ecosystems
Identification of driving variables	Identification of slow variables^7^	2	2.4	2.4 – Slow variables are acknowledged through agricultural practices that help maintain ecosystems, adapt to climate change and drought, and improve land and soil quality
		6	6.3	6.3 – Efforts to improve water quality and reduce water pollution acknowledge its role as a slow variable
		14	6.6	6.6 – Protection and restoration of water-related ecosystems acknowledge underlying slow variables, particularly wetlands, lakes, and aquifers
		15	14.1	14.1 – Aims at addressing marine pollution, which is a slow variable
			14.3	14.3 – Aims at addressing ocean acidification, which is a slow variable
			15.3	15.3 – Combating desertification and restoring degraded land and soil acknowledges these slow variables
	Identification of variables leading to tight feedbacks and coupling^4^			
Connectivity	Identification and degree of connectivity^8^			

References to the literature where our understanding of each resilience factor is developed are given as superscript numbers and the reference list can be found in the Supplementary material.

**Table 2.** List of social resilience factors that are addressed (at least in part) by SDG targets

Dimension	Resilience factor^reference^	SDGs	Targets	Rationale as to why the target aligns with the resilience factor
Governance	Heterogeneity and scale of governance (panarchy)^9^	6	6.5	6.5 – Integrated water resources at all levels, including transboundary where necessary, implies cognisance of heterogeneity and multi-level governance of river systems
	Presence of bridging organisations^10^			
	Presence of networks^11^	17	17.16	17.16 – Networks acknowledged in the aim to enhance multi-stakeholder partnerships that mobilise and share knowledge, expertise, technology, and financial resources
	Leadership (type and at what scales)^10^	17	17.15	17.15 – Emphasises respect for each country’s leadership to implement sustainable development policies
	Communication, information sharing^11^	2	2.3	2.3 – Access to knowledge as a means to increase agricultural productivity reflects information sharing
		3	2.5	2.5 – Equitable sharing of benefits from traditional knowledge reflects information sharing
		5	2.a	2.a – Enhancing international cooperation for agricultural research and development
		6	3.7	3.7 – Ensuring access to sexual and reproductive information and education
		7	3.b	3.b – Ensuring flexible access to intellectual property rights regarding medicines and information to protect public health
		9	5.b	5.b – Enhancing the use of information and communications technology for women
		12	6.5	6.5 – Integrated water resources management and transboundary cooperation imply communication and information sharing
		16	6.a	6.a – International cooperation for water and sanitation implies communication and information sharing
		17	7.a	7.a – Enhancing international cooperation for clean energy research and technology
			9.c	9.c – Increasing access to information and communications technology in least developed countries
			12.8	12.8 – To ensure people everywhere have relevant information for sustainable development
			16.10	16.10 – Aimed at ensuring public access to information
			17.6	17.6 – Enhances knowledge sharing
			17.7	17.7 – Promotes the dissemination and diffusion of technologies to developing countries
			17.8	17.8 – Enhances the use by LDCs of information and communication technology
			17.16	17.16 – To enhance partnerships that share knowledge
	Organisational learning^11^			
	Stakeholders and public involved in decision-making^12^	5	5.5	5.5 – Ensuring women’s full and effective participation in decision-making recognises their importance as stakeholders
		6	6.5	6.5 – Integrated water resources management and transboundary cooperation imply stakeholder involvement
		10	6.b	6.b – Participation of local communities in water and sanitation management
		11	10.2	10.2 – Promoting political inclusion of all
		16	10.6	10.6 – Enhancing representation and voice for developing countries in international decision-making
		17	11.3	11.3 – Enhancing participatory, integrated human settlement planning involves stakeholders
			16.7	16.7 – Responsive, inclusive, participatory, and representative decision-making at all levels
			16.8	16.8 – Participation of developing countries in global governance institutions
			17.17	17.17 – Promote effective public, public–private, and civil society partnerships
	Innovation^11^	8	8.3	8.3 – Promoting policies that support creativity and innovation
		9	9.5	9.5 – Encouraging innovation, research, and development in industrial sectors
		17	9.b	9.b – Ensuring policies that support industrial innovation in developing countries
			17.8	17.8 – Operationalise the innovation capacity-building mechanism for LDCs
	Polycentricity^13^			
Social capital	Diversity of livelihoods^14^	8	8.2	8.2 – Diversification of economic activity should translate to diversity of livelihoods
		15	15.c	15.c – Combat poaching and trafficking by increasing local capacity to pursue sustainable livelihoods
	Social modularity^15^			
	Local and organisational knowledge^15^	2	2.3	2.3 – Access to knowledge as a means to increase agricultural productivity requires social capital
		4	2.5	2.5 – Equitable sharing of benefits from traditional knowledge acknowledges social capital
		13	4.7	4.7 – Acquisition of knowledge for sustainable development
			13.3	13.3 – Improving climate change education and awareness enhances knowledge
	Trust among stakeholders^14^			
	Trust in government^14^	16	16.5	16.5 – Reduce corruption and bribery in all their forms
			16.6	16.6 – Effective, accountable, and transparent institutions at all levels
			16.7	16.7 – Responsive, inclusive, participatory, and representative decision-making at all levels
	Rule of law^16^	14	14.c	14.c – Conserve ocean resources through implementation of international law
		16	16.3	16.3 – Promote the rule of law at national and international levels
			16.b	16.b – Promote and enforce non-discriminatory laws
	Transparent and fair legal process^17^	16	16.3	16.3 – Ensure equal access to justice for all
			16.6	16.6 – Effective, accountable, and transparent institutions at all levels
			16.7	16.7 – Responsive, inclusive, participatory, and representative decision-making at all levels
	Transparent and fair policy process^17^	16	16.6	16.6 – Effective, accountable, and transparent institutions at all levels
			16.7	16.7 – Responsive, inclusive, participatory, and representative decision-making at all levels
	Robustness of institutions^18^	10	10.6	10.6 – Delivering more effective, credible, accountable, and legitimate institutions through inclusion
		16	16.6	16.6 – Effective, accountable, and transparent institutions at all levels
		17	16.a	16.a – Strengthen national institutions
			17.10	17.10 – Promote a universal, rules-based, open, non-discriminatory, and equitable multilateral trading system under the WTO
	Voluntary actions^14^			
Feedbacks amongst governance organisations and ecological change	Monitoring within a structured, iterative framework^19^	17	17.18	17.18 – Support developing countries to increase the availability of high-quality, timely, and reliable data
		*All*	17.19	17.19 – Develop measurements of progress on sustainable development *Follow-up and review inherent in the Agenda; monitoring conducted using the SDG indicators and reporting occurs at the High-Level Political Forum in Voluntary National Reviews*
	Ecosystem services used in decision-making^6^	8	8.4	8.4 – Endeavouring to decouple economic growth from environmental degradation acknowledges the use of ecosystem services in decision-making
		12	12.2	12.2 – Achieving sustainable management and efficient use of natural resources implies ecosystem services should be used in decision-making
		14	14.4	14.4 – Use of maximum sustainable yield and science-based management plans in fishing regulations
		15	15.9	15.9 – Integrating ecosystems and biodiversity into national and local planning

References to the literature where our understanding of each resilience factor is developed are given as superscript numbers and the reference list can be found in the Supplementary material.

**Table 3.** List of economic resilience factors that are addressed (at least in part) by SDG targets

Dimension	Resilience factor^reference^	SDGs	Targets	Rationale as to why the target aligns with the resilience factor
Diversity	Within scale diversity^1^	8	8.2	8.2 – Diversification of economic activity
		9	9.b	9.b – Ensuring policies that support industrial diversification
	Cross-scale redundancy^1^			
	Response diversity^2^	8	8.2	8.2 – Diversification of economic activity
		9	9.b	9.b – Ensuring policies that support industrial diversification
		14	14.7	14.7 – Increasing diverse economic benefits of oceans to SIDS and LDCs through sustainable fisheries, aquaculture, and tourism
	Diversity of employment^20^	8	8.2	8.2 – Diversification of economic activity
		14	14.7	14.7 – Increasing diverse economic benefits of oceans to SIDS and LDCs through sustainable fisheries, aquaculture, and tourism
	Diversity of firms^20^	8	8.2	8.2 – Diversification of economic activity
Innovation	Ability to take risk^21^	8	8.3	8.3 – Promoting policies that support creativity and innovation should enhance ability to take risk
		10	8.10	8.10 – Encouraging financial institutions to expand access to banking, insurance, and financial services should enhance ability to take risk
			10.c	10.c – Reducing transaction costs of migrant remittances could enhance ability of migrants to take risk
	Access to financial capital^22^	1	1.4	1.4 – Ensuring access to economic resources, new technology, and financial services
		2	2.3	2.3 – Ensuring access for small-scale farmers to financial services and productive resources
		3	3.c	3.c – Increase health financing particularly in least developed countries
		5	5.a	5.a – Ensuring equal access for women to financial resources and services
		8	8.3	8.3 – Policies that support innovation and enterprise creation through access to financial services
		9	8.10	8.10 – Encouraging financial institutions to expand access to banking and financial services
		10	9.3	9.3 – Increasing access of small-scale industrial enterprises to financial services and affordable credit
		11	9.a	9.a – Enhancing financial support to developing countries for infrastructure development
		13	10.b	10.b – Encouraging financial flows to States in greatest need
		15	11.c	11.c – Providing financial assistance to least developed countries for buildings
		17	13.a	13.a – Mobilising and operationalising the Green Climate Fund
			15.a	15.a – Mobilising financial resources to conserve and sustainably use biodiversity and ecosystems
			15.b	15.b – Mobilising financial resources for sustainable forest management
			17.1	17.1 – Improving domestic capacity for tax and other revenue collection through international support
			17.2	17.2 – Implementation of official development assistance commitments from developed countries
			17.3	17.3 – Mobilisation of additional financial resources for developing countries
			17.4	17.4 – Debt financing, relief, and restructuring for developing countries
	Regulatory and market-based incentives^23^	2	2.b	2.b – Regulatory measures to correct and prevent trade distortions global agricultural markets
		10	2.c	2.c – Measures to ensure properly functioning food markets
		12	10.5	10.5 – Improving the regulation and monitoring of global financial markets
		14	10.a	10.a – Implementing special and differential treatment for developing countries in global trade
		15	12.c	12.c – Phasing out harmful subsidies, removing market distortions around fossil-fuels, and restructuring taxation to reflect environmental impacts
		17	14.4	14.4 – Regulation of fishing
			14.6	14.6 – Removal of harmful subsidies that lead to illegal or overfishing
			14.b	14.b – Providing small-scale fishers access to markets
			15.b	15.b – Providing adequate incentives for developing countries to pursue sustainable forest management
			17.12	17.12 – Implement duty-free and quota-free market access for LDCs
	Reporting and information requirements^24^	12	12.6	12.6 – Encouraging companies to include sustainability information in their reporting cycle
		17	17.18	17.18 – Support developing countries to increase the availability of high-quality, timely, and reliable data
		*All*	17.19	17.19 – Develop measurements of progress on sustainable development *Follow-up and review inherent in the Agenda; monitoring conducted using the SDG indicators and reporting occurs at the High-Level Political Forum in Voluntary National Reviews*
	Planning requirements^25^	11	11.3	11.3 – Enhancing participatory, integrated, and sustainable human settlement planning
		13	11.a	11.a – Strengthening national and regional development planning
		15	13.2	13.2 – Integrating climate change measures into planning
			13.b	13.b – Promoting climate change planning in least developed countries and small islands
			15.9	15.9 – Integrating ecosystems and biodiversity into national and local planning
	Disclosure^26^			

References to the literature where our understanding of each resilience factor is developed are given as superscript numbers and the reference list can be found in the Supplementary material.

## Data Availability

All data are available in Tables 1–3. Figures are produced by counting and compiling the data in Tables 1–3.
