# Supplementary material for: Towards a global sustainable development agenda built on social–ecological resilience
Source: Glob Sustain. Author manuscript; Available in PMC 2024 Apr 24. (PMC10489559; doi:10.1017/sus.2023.8)
Supplement: SI [file NIHMS1918853-supplement-SI.docx]

**Supplementary Information**

**Towards a global sustainable development agenda built on social-ecological resilience**

Murray W. Scown, Robin K. Craig, Craig R. Allen, Lance Gunderson, David G. Angeler, Jorge H. Garcia and Ahjond Garmestani

**Reference list for Tables 1–3**

1. Peterson, G.D., Allen, C.R. and Holling, C.S. 1998. Ecological resilience, biodiversity, and scale. *Ecosystems* 1: 6-18.
2. Elmqvist, T., Folke, C., Nystrom, M., Peterson, G.D., Bengtsson, J., Walker, B.H., Norberg, J. 2003. Response diversity, ecosystem change, and resilience. *Frontiers in Ecology and the Environment* 1: 488-494.
3. Allen, C.R., D.G. Angeler, G.S. Cumming, C. Folke, D. Twidwell and D.R. Uden. 2016. Quantifying spatial resilience. *Journal of Applied Ecology* 53: 625-635.
4. Gunderson, L., C. R. Allen, and D. Wardwell. 2007. *Temporal scaling in complex systems: resonant frequencies and biotic variability*. Chapter 5 in Bissonette, J. A., and I. Storch, eds. Temporal dimensions in landscape ecology: wildlife responses to variable resources. Springer.
5. Walker, B., C. S. Holling, S. R. Carpenter, and A. Kinzig. 2004. Resilience, adaptability and transformability in social–ecological systems. *Ecology and Society* 9(2): 5.
6. Birge, H.E., C.R. Allen, A.S. Garmestani and K.L. Pope. 2016. Adaptive management for ecosystem services. *Journal of Environmental Management* 183: 343-352.
7. Holling, C.S. 1973. Resilience and stability of ecological systems. *Annual Review of Ecology and Systematics* 4: 1-23.
8. Chambers, J.C., C.R. Allen and S.A. Cushman. 2019. Operationalizing ecological resilience concepts for managing species and ecosystems at risk. *Frontiers in Ecology and Evolution* 7: 241.
9. Garmestani, A.S. and M.H. Benson. 2013. A framework for resilience-based governance of social-ecological systems. *Ecology and Society* 18 (1): 9.
10. Folke, C., T. Hahn, P. Olsson and J. Norberg. 2005. Adaptive governance of social-ecological systems. *Annual Review of Environment and Resources* 30: 441-473.
11. Chaffin, B.C., A.S. Garmestani, L.H. Gunderson, M.H. Benson, D.G. Angeler, C.A. Arnold, B. Cosens, R.K. Craig, J.B. Ruhl and C.R. Allen. 2016. Transformative environmental governance. *Annual Review of Environment and Resources* 41: 399-423.
12. Folke, C., Biggs, R., Norström, A. v, Reyers, B., Rockström, J., 2016. Social-ecological resilience and biosphere-based sustainability science. *Ecology and Society* 21.
13. Ostrom, E. 2010. A long polycentric journey. *Annual Review of Political Science* 13: 1-23.
14. Adger, W.N. 2003. Social capital, collective action, and adaptation to climate change. *Economic Geography* 79: 387-404.
15. Ostrom, E. 2007. A diagnostic approach for going beyond panaceas. *Proceedings of the National Academy of Sciences* 104: 15181-15187.
16. Garmestani, A.S. and C.R. Allen. 2014. *Social-Ecological Resilience and Law*. New York: Columbia University Press.
17. Craig, R.K., A.S. Garmestani, C.R. Allen, C.A. Arnold, H. Birgé, D.A. DeCaro, A.K. Fremier, H. Gosnell and E. Schlager. 2017. Balancing stability and flexibility in adaptive governance: an analysis of tools available in U.S. environmental law. *Ecology and Society* 22(2):3.
18. Anderies, J. M., M. A. Janssen, and E. Ostrom. 2004. A framework to analyze the robustness of social-ecological systems from an institutional perspective. *Ecology and Society* 9(1): 18.
19. Herrmann, D.L., K. Schwarz, C.R. Allen, D.G. Angeler, T. Eason and A. Garmestani. 2021. Iterative scenarios for social-ecological systems. *Ecology and Society* 26(4):8.
20. Garmestani, A.S., C.R. Allen, J.D. Mittelstaedt, C.A. Stow and W.A. Ward. 2006. Firm size diversity, functional richness and resilience. *Environment and Development Economics* 11: 533-551.
21. Mazzucato, M. 2011. *The Entrepreneurial State*. London: Demos
22. Stiglitz, J.E. 1985. [Credit Markets and the Control of Capital](https://ideas.repec.org/a/mcb/jmoncb/v17y1985i2p133-52.html). [*Journal of Money, Credit and Banking*](https://ideas.repec.org/s/mcb/jmoncb.html) 17: 133-152
23. Garcia, J. H., and Sterner, T. 2021. Carbon Taxes. Oxford Research Encyclopedia of Environmental Science. doi:10.1093/acrefore/9780199389414.013.727
24. Afsah S, Blackman A, Garcia JH, Sterner T. 2013. *Environmental Regulation and Public Disclosure: The Case of PROPER in Indonesia?* Routledge
25. Sterner, T., and J. Coria. 2012. *Policy instruments for environmental and natural resource management* (2nd ed.). RFF Press.
26. Garcia J.H., Afsah S. and T. Sterner. 2009. What types of firms are more sensitive to public disclosure schemes for pollution control? Evidence from Indonesia. *Environmental and Resource Economics* 42: 141-168
